# The Role of Stress-Induced O-GlcNAc Protein Modification in the Regulation of Membrane Transport

**DOI:** 10.1155/2017/1308692

**Published:** 2017-12-31

**Authors:** Viktória Fisi, Attila Miseta, Tamás Nagy

**Affiliations:** ^1^Department of Laboratory Medicine, School of Medicine, University of Pécs, Ifjúság str. 13, Pécs 7624, Hungary; ^2^János Szentágothai Research Centre, University of Pécs, Ifjúság str. 13, Pécs 7624, Hungary

## Abstract

O-linked N-acetylglucosamine (O-GlcNAc) is a posttranslational modification that is increasingly recognized as a signal transduction mechanism. Unlike other glycans, O-GlcNAc is a highly dynamic and reversible process that involves the addition and removal of a single N-acetylglucosamine molecule to Ser/Thr residues of proteins. UDP-GlcNAc—the direct substrate for O-GlcNAc modification—is controlled by the rate of cellular metabolism, and thus O-GlcNAc is dependent on substrate availability. Serving as a feedback mechanism, O-GlcNAc influences the regulation of insulin signaling and glucose transport. Besides nutrient sensing, O-GlcNAc was also implicated in the regulation of various physiological and pathophysiological processes. Due to improvements of mass spectrometry techniques, more than one thousand proteins were detected to carry the O-GlcNAc moiety; many of them are known to participate in the regulation of metabolites, ions, or protein transport across biological membranes. Recent studies also indicated that O-GlcNAc is involved in stress adaptation; overwhelming evidences suggest that O-GlcNAc levels increase upon stress. O-GlcNAc elevation is generally considered to be beneficial during stress, although the exact nature of its protective effect is not understood. In this review, we summarize the current data regarding the oxidative stress-related changes of O-GlcNAc levels and discuss the implications related to membrane trafficking.

## 1. Introduction

The function and impact of protein O-linked N-acetylglucosamine (O-GlcNAc) modification are very complex and only partially discovered despite almost 1300 scientific studies were published in the last 30 years. Although a relatively simple molecular mechanism (the addition and removal of N-acetylglucosamine on Ser/Thr residues), it is a focal point of numerous converging and diverging cellular events. One of the most profound properties of O-GlcNAc is that it is directly embedded in the metabolic regulation of the cells [1]. Since metabolism is basically required for and influences every other cellular function, the role of O-GlcNAc to mediate signals to and from metabolic systems seems to be an obvious choice for nature. Indeed, several studies showed that both increased (e.g., in diabetes) and decreased (fasting) glucose metabolism have an impact on O-GlcNAc [2–6]. Consequently, O-GlcNAc directly influences various regulatory systems, such as the transcriptional machinery, protein synthesis, trafficking and degradation, and regulation of glucose uptake [7, 8]. Glucose metabolism was the most studied element in this respect, but the involvement of other metabolic pathways (nucleotide synthesis, amino acid, and lipid metabolism) was also proposed [1].

Despite difficulties in discerning cause and effect (e.g., malignant cells may develop altered metabolic rate and/or O-GlcNAc levels independently), it appears that besides metabolic challenges, regular cellular events such as mitosis, cell differentiation, and response to a hormonal signal or cell-cell adhesion may also directly influence O-GlcNAc modifications on proteins [9–13]. Moreover, a wide variety of stressors, including osmotic challenge, hyperthermia, heavy ion toxicity, hypoxia, and oxidative stress, was also shown to impact O-GlcNAc [14–17]. The most comprehensive data available were provided by studies done on cardiomyocytes under ischemic or oxidative conditions. The majority of these studies showed that elevation of O-GlcNAc prevented or at least ameliorated the damage caused by the stress. Several mechanisms were proposed to explain the protective effect of O-GlcNAc under stress situation, such as increased heat shock protein synthesis, inhibition of protein degradation, inhibition of apoptosis, and modulation of calcium homeostasis [18]. Although membrane transport is involved in many of these mechanisms and several studies demonstrated evidences concerning O-GlcNAc's influence on membrane trafficking (Table 1), a comprehensive understanding of this interaction is missing. In this review, we summarize our current understanding of the intracellular process called O-GlcNAc modification, its adaptive response regarding oxidative stress, and its influence on membrane traffic, including glucose and ion transport and also synaptic, nuclear, and mitochondrial transport.

## 2. Regulation of Proteins by O-GlcNAc

O-linked N-acetylglucosamine (or O-GlcNAc) is a reversible, dynamic posttranslational modification (PTM) affecting serine and threonine residues of proteins. It was first discovered in 1984 by Torres and Hart [19]. The set of O-GlcNAc targets includes around 1500 proteins which are located both in the nucleus, the cytoplasm, and the mitochondria of the cells [20]. Likewise, several membrane proteins were found to be O-GlcNAcylated on their intracellular domain, for example, inositol 1,4,5-trisphosphate (InsP3) receptor type I, beta-amyloid precursor protein (APP), or epidermal growth factor receptor (EGFR) [21–23]. Interestingly, recent discoveries showed that even extracellular domains can carry the O-GlcNAc modification [24]; however, the latter seems to be irreversible and controlled by a different enzyme (EGF repeat-specific O-GlcNAc transferase termed EOGT) than cytoplasmic O-GlcNAc modification.

**Figure 1:**
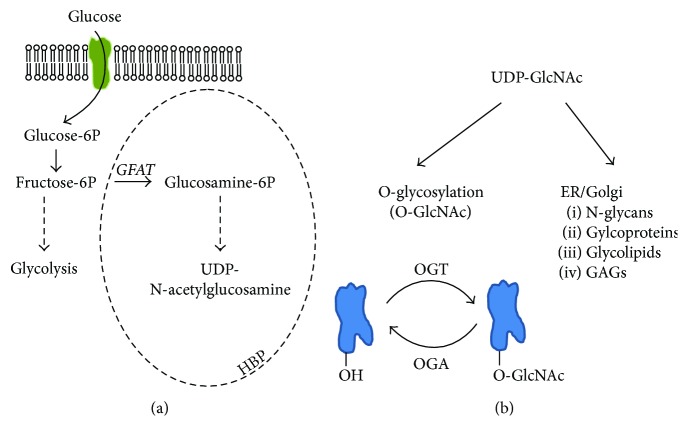
The hexosamine biosynthesis pathway (HBP) and the O-GlcNAc posttranslational modification. (a) An estimated 1–3% of the total glucose enters HBP. The key enzymatic reaction of this pathway is the addition of an amino group from glutamine to fructose-6-phosphate by the rate-limiting enzyme glutamine-fructose-6-phosphate amidotransferase (GFAT). Following subsequent steps (addition of an acetyl group, converting 6-phosphate to 1-phosphate and finally the transfer to UDP), the end product of HBP is uridine diphosphate N-acetylglucosamine (UDP-GlcNAc). (b) The majority of UDP-GlcNAc is utilized in the endoplasmic reticulum and the Golgi apparatus for various glycolipid, glycoprotein, and glycan synthesis. A small, but significant, percentage of UDP-GlcNAc serves as a substrate pool for the dynamic, reversible posttranslational modification termed O-GlcNAc. A single N-acetylglucosamine group is attached to the Ser/Thr residues of target proteins by O-GlcNAc transferase, while the removal of this group is managed by the enzyme O-GlcNAcase. O-GlcNAc modification occurs predominantly in the cytoplasm and in the nucleus, and it is strongly dependent on substrate availability (i.e., the metabolic flux through HBP).

O-GlcNAc cycling is controlled by the action of O-GlcNAc transferase (OGT) and O-GlcNAcase (OGA), the enzymes that add and remove O-GlcNAc, respectively. The substrate of the transferase reaction is the uridine diphosphate N-acetylglucosamine (UDP-GlcNAc), the product of the nutrient-sensitive hexosamine biosynthetic pathway (HBP). Approximately, 1–3% of total glucose is entering this pathway which integrates glucose, amino acid, fatty acid, and nucleotide metabolism [1]. Using UDP-GlcNAc, OGT attaches a single O-linked N-acetylglucosamine (O-GlcNAc) moiety to Ser or Thr residues of proteins (Figure 1).

O-GlcNAc modification may influence the proteins' functions [7, 25, 26], protect from degradation [27, 28], influence protein-protein interaction or localization [29–32], and possibly alter protein hydrophobicity [17]. The most studied effect is its competition with phosphorylation, since O-GlcNAc can occupy the same residues as phosphorylation. However, other interactions such as proximal site competition and proximal site occupation were also proposed [25]. O-GlcNAc also influences protein synthesis via modulation of the action of transcription factors such as c-myc, NF*κ*B, and p53 [33–37]. It is estimated that around 25% of the O-GlcNAc-modified proteins are involved in transcriptional regulation [38]. Mechanistically, O-GlcNAcylation can affect the translocation, DNA binding, transactivation and stability of transcription factors. Moreover, O-GlcNAcylation regulates protein synthesis also by cotranslational glycosylation which protects nascent polypeptide chains from ubiquitination [28]. This interplay with ubiquitination also has a general impact on protein stability and turnover by reducing proteasome degradation [27, 39]. O-GlcNAc plays a role in protein folding and unfolded protein response as well [40, 41].

O-GlcNAc modification is abundantly present in higher eukaryotes, and it is required for the normal functions of the cells [42, 43]. It influences several cellular processes, including nutrient sensing, cell cycle regulation, transcriptional regulation, Ca^2+^ handling, cytoskeletal organization, or nuclear translocation [1, 7, 25, 44–48]. Naturally, O-GlcNAc's involvement in pathophysiological processes was soon proposed. Its reciprocal relationship with phosphorylation was best characterized in Alzheimer's models; abnormal low level of O-GlcNAc may give a way for hyperphosphorylation on tau proteins which are prone to form neuron-damaging neurofibrillary tangles [49, 50]. Elevated level of O-GlcNAc can also cause deleterious effects: in diabetes, long-term hyperglycemia will inevitably lead to increased flux through the HBP and increased O-GlcNAc [51, 52]. Chronic imbalance of O-GlcNAc could lead to disturbed transcriptional factor activation, reactive oxygen species production, altered signal transductions, or inhibition of eNOS activity [53, 54]. Surprisingly, a few studies found that short-term hypoglycemia also elevated O-GlcNAc levels [6]. This paradox may be resolved if O-GlcNAc is considered as a stress adaptation mechanism that is triggered by acute challenges, such as hypoglycemia. Indeed, a large number of data suggest that protein O-GlcNAc modification dynamically increases after the cells are exposed to various type of environmental challenges [15, 16].

## 3. Oxidative Stress and O-GlcNAc

Disruption of redox regulation has been implicated in many conditions, such as aging, neurodegenerative diseases, ischemic events, arterial hypertension, and diabetes. Recent advances in O-GlcNAc-related studies suggest that disturbed O-GlcNAc regulation is involved in the development of these conditions. It also seems to be that stress response and O-GlcNAc are connected [14, 55]. Increasing number of evidences suggest that oxidative stress may stimulate the hexosamine biosynthetic pathway and consequently O-GlcNAcylation [16, 51]. Reactive oxygen species (ROS) can modify protein functions by oxidation of cysteine residues [56]. Within the glycolytic metabolic pathway, the activity of glyceraldehyde 3-phosphate dehydrogenase (GAPDH) is particularly sensitive to inhibition by ROS [57]. GAPDH is a key enzyme controlling the metabolic flux through glycolysis and Krebs cycle; thus, its inhibition results in diverting glucose to bypass pathways, such as the pentose phosphate shunt and the HBP [14, 51]. It has also been shown that inhibition of GAPDH by mitochondrial superoxide is an important factor in increased O-GlcNAcylation associated with hyperglycemia [54, 58]. Jones et al. have shown in myocytes that incubation with hydrogen peroxide caused an early increase of O-GlcNAc levels followed by a gradual decrease after 45 min [59]. Similarly, ROS-induced O-GlcNAcylation has been reported in cultured 3T3-L1 adipocytes. In these cells, urea induced ROS production which increased O-GlcNAc modification of insulin signaling molecule IRS-1 [60]. Kátai et al. demonstrated that protein O-GlcNAcylation was transiently elevated in a neuroblastoma cell line following oxidative stress [16]. The expressional level or the activity of OGT after various stresses, including hypoxia/reperfusion and oxidative stress, has been also found to be increased [15, 16, 61]. Interestingly, in some of the studies, the activity and expression of OGA also increased following stress [62].

O-GlcNAc can in turn modulate the response to oxidative stress: however, data are contradictory in this field [14]. It has been shown that increased O-glycosylation by either elevated OGT activity or OGA inhibition attenuated ROS generation induced by H_2_O_2_ or hypoxia [63–65]. However, Goldberg et al. demonstrated that high-glucose-induced ROS production was prevented by O-GlcNAc depletion in mesangial cells, which was speculated to be caused by the influence of O-GlcNAc on NADPH oxidase phosphorylation [66]. Nevertheless, the majority of the data shows that O-GlcNAc either directly or indirectly influences enzymes participating in the redox regulation [67]. For example, in various tissues, hyperglycemia induces an increase in mitochondrial superoxide production in association with elevated O-GlcNAcylation and a reciprocal decrease in phosphorylation of eNOS at the primary positive regulatory site, Ser-1177 [54, 68, 69]. Inducible NOS (iNOS) production is mediated by the NF*κ*B pathway, of which stimulation by TNF*α* or LPS has been reported to be affected by O-GlcNAc. TNF*α*-induced iNOS expression was shown to be drastically decreased by high O-GlcNAc [70, 71], while LPS-induced NF-*κ*B activation is suggested to be inhibited by OGT [72]. Expression of endogenous enzymatic antioxidants like superoxide dismutase, glutathione peroxidase, and catalase is increased in the case of OGA inhibition, while reduction of O-GlcNAc decreases expressional level of these genes [63, 73].

An important mediator of response to oxidative stress is the heat shock protein (HSP) family. HSP expression is at a low level under normal physiological conditions, but in response to stress such as heat, oxidative damage, or heavy metal poisoning, it dramatically increases [74]. Zachara et al. found that elevating O-GlcNAc by blocking OGA prior to heat shock increased the thermotolerance and sped up the increase of HSP70 and HSP40 [15]. In a later article, the same research group also showed that O-GlcNAc modification might compete with glycogen synthase kinase 3*β*-dependent phosphorylation (GSK3*β*) on HSP90*β* proteins [75]. Using an inducible OGT null cell line, Kazemi et al. have screened the expression of 84 molecular chaperones and have shown that 18 HSP proteins have reduced mRNA expression. The authors found that O-GlcNAc may also influence HSP expression by promoting the phosphorylation and inhibition of GSK-3*β* [76].

In general, augmentation of O-glycosylation seems to attenuate oxidative damage. The connection between O-GlcNAc and oxidative stress was extensively studied in neural, retinal cells and cardiomyocytes due to its clinical significance in neurodegenerative disorders and ischemic organ damages [16, 63, 77, 78]. In response to ischemia-reperfusion injury, the amount of O-GlcNAc dramatically increases [64, 79, 80]. The role of O-GlcNAc in myocardial protection against oxidative stress is associated with calcium paradox. It is suggested that elevated HBP flux and O-GlcNAc inhibit Ca^2+^ influx [45]. Changes in intracellular Ca^2+^ play a critical role in initiating cardiomyocyte apoptosis and necrosis resulting from Ca^2+^ overload [79].

## 4. O-GlcNAc and Ion Channels

### 4.1. Ca^2+^ Channels

While intracellular free calcium ([Ca^2+^]_i_) concentration remains usually below 100 nM [81], in extracellular space and in compartmentalized intracellular stores, the calcium concentration is 10000 times higher, up in the millimolar range. [Ca^2+^]_i_ is regulated by a multitude of mechanisms, specific channels allowing Ca^2+^ to enter the cells from the extracellular space such as voltage-gated channels, ligand-gated channels, and the elusive store-operated channels. Ryanodine receptors and IP3 receptors present on the endoplasmic reticulum or sarcoplasmic reticulum can also quickly release a large amount of Ca^2+^. The removal of [Ca^2+^]_i_ is managed mostly by SERCA, the plasma membrane Ca^2+^-ATPase (PMCA), and Na^+^/Ca^2+^ exchanger (NCX) [82, 83]. Calcium-binding proteins, such as calmodulin, calcineurin, and also the actin-myosin-troponin complex, are not only downstream elements of the [Ca^2+^]_i_ signaling but also serve as intracellular buffer to bind Ca^2+^ [84]. Intracellular calcium signaling is similarly versatile compared to O-GlcNAc regulation; [Ca^2+^]_i_ is a central messenger in several signaling pathways, influencing transcriptional factors, calcium-dependent phospholipase A2, protein kinase C, and various proteases [85]. It is a significant contributor to the deleterious effects of ischemia/reperfusion injuries, that is, abnormal elevation of [Ca^2+^]_i_ will lead to apoptosis and hypertrophy [63, 86, 87].

O-GlcNAc and [Ca^2+^]_i_ regulation can intercept each other at several levels. Many of the downstream elements of [Ca^2+^]_i_ signaling are influenced by O-GlcNAc modification, including calmodulin-dependent kinase IV, myosin, actin, and PKC [88–90]. More importantly, O-GlcNAc seems to be influencing [Ca^2+^]_i_ as well [45]. The most studied models in this respect were cardiac ischemia/reperfusion and calcium paradox experiments [65, 79]. Based on these studies, it seems to be that artificially elevating O-GlcNAc levels are a prosurvival mechanism [91]. Moreover, it was found that stress itself will elevate O-GlcNAc and that preconditioning protects the cells at least partially via increased O-GlcNAc levels [92]. One of the mediators of this protection was [Ca^2+^]_i_. O-GlcNAc was shown to suppress calcium elevation and calcium overload elicited by agonists, oxidative stress. Liu et al. showed that increased O-GlcNAc is also effective to decrease calcium overload in calcium-paradox experiments when a short perfusion with Ca^2+^-free medium followed by perfusion with a normal amount of Ca^2+^ would lead to rapid calcium overload and cellular damage [79].

Which of the [Ca^2+^]_i_ regulatory elements are affected by O-GlcNAc? Regarding voltage-dependent calcium channels, detailed functional information is not yet available but Trinidad et al. identified calcium voltage-gated channel subunit beta 3 (CACNB3), gamma 3 (CACNG3), alpha 1B (CACNA1B), alpha 1A subunit (CACNA1A), and alpha 1G subunit (CACNA1G) as O-GlcNAc modified proteins of murine synapse [93]. SERCA itself has been described to be O-GlcNAc modified [94], while O-GlcNAcylation of phospholamban (SERCA repressor) also modulates its inhibitory effects on SERCA, correlating with reduced cardiac function in diabetic cardiomyopathy [95]. A frequently studied nonvoltage-gated calcium entry pathway is the store-operated calcium entry (SOCE) which is an extracellular Ca^2+^ influx into the cytoplasm in response to intracellular Ca^2+^ store depletion. Nagy et al. demonstrated that the latter process is blocked by O-GlcNAc elevation in cardiomyocytes [45]. Although the exact mechanism of SOCE still needs to be clarified, another important mediator protein of SOCE, stromal interaction molecule 1 (STIM1), has been proved to be influenced by O-GlcNAc [96]. During ER Ca^2+^ depletion induced by thapsigargin or EGTA, STIM1 proteins form puncta in ER/SR membrane which was inhibited in a dose-dependent manner by elevating O-GlcNAc levels. Moreover, it has been shown that STIM1 itself is a target for O-GlcNAc and that increasing STIM1 O-GlcNAcylation significantly modified its phosphorylation [96].

### 4.2. Other Ion Channels

Given that calcium is an important intracellular messenger, it is no surprise that its interaction with O-GlcNAc modification attracted significant research interest. In contrast to calcium, the influence of O-GlcNAc on the regulation of other ions and ion channels is less documented yet. Nevertheless, the data available at present does suggest that O-GlcNAc—and consequently O-GlcNAc-related cellular mechanisms such as carbohydrate metabolism and/or stress response—might have an impact on several elements of sodium, potassium, and chloride transport. For example, Ruan et al. have shown that voltage-dependent K^+^ channel KCNQ3 (Kv7.3) interacts with OGT and it is probably O-GlcNAc modified at threonine 655 in neurons [97]. This protein functions in the regulation of neuronal excitability by associating with the related KCNQ2 or KCNQ5 thus forming an M-channel [98]. Defects in this gene are a cause of benign familial neonatal convulsions (BFNC) [99]. KCNN2, KCNN3, KCNMA1, and KCNA4 were also identified by mass spectrometry screening as potential O-GlcNAc proteins [93].

To our knowledge, no direct evidence was published yet on the potential O-GlcNAc modification of sodium channels. However, indirect data shows that sodium pumps still might be influenced by O-GlcNAc. The same study that probed murine synapses for O-GlcNAc-modified proteins and identified potassium channels also found HexNAc peptide characteristic for voltage-gated sodium channels and for sodium/potassium-transporting ATPases [93]. Indirect influence by O-GlcNAc on sodium transport has been speculated by other authors. Namely, isoforms of ankyrin G at nodes of Ranvier have been shown to be modified by O-GlcNAc. Ankyrins are spectrin-binding proteins that link the cytoplasmic domains of membrane proteins to the spectrin/actin network, in particular, ankyrin G binds voltage-gated sodium channels. The authors speculate that O-GlcNAcylated serine-rich domain of ankyrin G may be involved in targeting voltage-dependent sodium channels to specific locations [100].

Among voltage-dependent anion channels (VDACs), VDAC1 seems to be O-GlcNAc modified [59, 93]. VDAC1 is extensively studied because of its clinical significance. It was shown to be overexpressed in many cancer types, and silencing of its gene inhibits tumor growth [101]. VDAC plays a critical role in the transport of small, negatively charged molecules across the mitochondrial membrane. Considering that this function is an important part of the cellular metabolism, it is plausible that O-GlcNAc modification could regulate mitochondrial activity through VDAC. Indirect evidence shows that Cl^−^ channels might be also regulated by O-GlcNAc. For example, the expression of chloride channel-2 (ClC-2) is dependent on the Sp1 transcription factor. Vij and Zeitlin published that O-GlcNAcylation of Sp1 is required for proper ClC-2 gene expression [102]. On the other hand, our recent publication [17] showed that osmotic resistance and volume regulation are influenced by O-GlcNAc. Since osmotic regulation (under hypotonic conditions) is controlled in a large part by a mechanism called regulatory volume decrease and carried out by the activation of a chloride conductance upon cell swelling (IClswell). Based on bioinformatics analysis of the ICln protein, which is responsible for IClswell, the presence of various O-GlcNAcylation sites on ICln is likely [103].

### 4.3. Glucose Transport

There is plenty of evidence that O-GlcNAc may regulate glucose transport. In fact, one of the first functions proposed for O-GlcNAc was nutrient sensing and O-GlcNAc is considered a key participant in insulin resistance [1]. As mentioned earlier, substrate production for O-GlcNAc is provided by the HBP. Since O-GlcNAc formation is dependent on the metabolic flux through HBP, which in turn is dependent on glucose (and subsequent fructose-6-phosphate) availability, metabolic changes can influence O-GlcNAc levels. Increased O-GlcNAc is thought to regulate glycogen synthesis, glucose metabolism, and glucose transport [5, 75, 104]. Several intermediate messengers of the insulin receptor signaling cascade have been identified as target for O-GlcNAc modulation such as IRS-1 and Akt [105–107].

The principal glucose transporter protein that mediates glucose uptake is glucose transporter type 4 (GLUT4), which plays a key role in regulating glucose homeostasis. GLUT4 is one of 13 sugar transporter proteins (GLUT1 to GLUT12 and HMIT) in humans. It is mainly expressed in skeletal muscle and adipose tissues. In an unstimulated state, it is mostly located in intracellular vesicles but a rapid translocation into the plasma membrane occurs after insulin stimulation to increase glucose uptake [108]. There is a growing evidence that increased O-GlcNAcylation of GLUT4 vesicle proteins such as Munc18c and others has a role in the inhibition of glucose transport in diabetes [109] and GLUT4 itself is suspected to be an O-GlcNAc target [110]. In cancer cells, the transcription factor HIF-1*α* (hypoxia-inducible factor 1*α*) induces a metabolic shift to aerobic glycolysis through the upregulation of various glycolytic proteins, including GLUT1 [111]. Ferrer et al. showed that OGT and O-GlcNAc modification are required to prevent HIF-1*α* proteasomal degradation in breast cancer cells thus enabling GLUT1 expression, glucose uptake, and survival in breast cancer cells [111].

### 4.4. Synaptic Transport

Nerve terminals are especially enriched in O-GlcNAcylation [112]. According to the recent data of Lagerlöf et al., OGT is present not only in presynaptic, but also in postsynaptic density and they proposed that O-GlcNAc is an important regulator of the synaptic maturation and plasticity [113]. One of the more abundant phosphoproteins in the brain is synapsin I. It belongs to the synapsin family that anchors synaptic vesicles to the cytoskeleton thus playing a role in neurotransmitter release control [114]. Synapsin I controls the size and release of residual pool of synaptic vesicles, and disruption of synapsin I function causes reduced size of the synaptic vesicle pool, defects in synaptic plasticity, memory deficits, and epileptic seizures. Synapsin I was among the first proteins that were found to be heavily O-GlcNAc modified [115]. Skorobogatko et al. have also found that synapsin I is O-GlcNAcylated during hippocampal synaptogenesis in rats. The authors identified three novel O-GlcNAc sites on the protein; two of them are also known as Ca^2+^/calmodulin-dependent protein kinase II phosphorylation sites. They also showed that the O-GlcNAc site of Thr-87—which is located within an amphipathic lipid-packing sensor motif—interferes with the binding of synapsin I to synaptic vesicles. When O-GlcNAc modification is impossible due to mutation of Thr-87, synapsin I tends to localize to synapses. Lacking O-GlcNAc on Thr-87 also led to increased density and size of synaptic vesicles [32]. Among with synapsin I, the protein Piccolo was also found to be heavily O-GlcNAc modified [116]. Both proteins are involved in the regulation of synaptic vesicles, and both are known to be phosphorylated, suggesting that interaction between O-GlcNAc and phosphorylation might have a mutual regulatory role.

Alpha-synuclein, a small protein consisting of 140 amino acids, is also implicated to take part in regulating neurotransmitter release. The protein is specifically enriched in presynaptic nerve terminals and is likely to play a role in the development of Parkinson's disease [117]. Its potential implication is suggested in exocytic processes and in the recycling of synaptic vesicles through association with the cell membrane [118]. In vivo, endogenous O-GlcNAcylation of alpha-synuclein at threonine 64 and 72 in mice and serine 87 in humans has been identified. Available data suggest that the presence of these modifications reduces the chance for aggregation and the toxicity of the protein but likely has no or little effect on its binding or remodeling membranes [118].

In a proteome-wide identification of O-GlcNAc-modified proteins, synergin gamma has also been detected as a target [37]. Through interaction with adaptor protein 1 (AP-1) complex, synergin gamma is involved in the trafficking of clathrin-coated vesicles to different directions like the trans-Golgi network or the plasma membrane [119, 120]. Perez-Cervera et al. have shown a connection between O-GlcNAc and lipid rafts. They demonstrated that OGT is present in lipid microdomains and that its localization at the raft is regulated by insulin signaling [121]. Although the role of O-GlcNAc is not clarified yet, it is noteworthy to mention that lipid rafts seem to play an important role in synaptic signaling and plasticity and, moreover, are involved in endocytic and exocytic transport routes [122, 123].

The O-GlcNAc modification of amyloid precursor protein (APP) carries special importance; inadequate proteolysis of APP produces amyloid-beta, which is a hydrophobic peptide and the major hallmark of Alzheimer's disease (AD) [22, 124]. APP is an integral membrane protein involved in synaptic formation and repair. While it is not known whether O-GlcNAc directly regulates the normal function of APP, its role in the normal processing and trafficking to the plasma membrane has been studied by several researchers [125–127]. Apparently, increased O-GlcNAc modification on APP will facilitate its traffic to the membrane and decrease its endocytosis, resulting in reduced formation of the pathological amyloid-beta products. This process could be a promising therapeutic target to be exploited. On the other hand, O-GlcNAc modification of APP has interesting implications regarding AD and carbohydrate metabolism. Type 2 diabetes is associated with a higher risk of AD, some even use the term “type 3 diabetes” for AD [128]. It seems to be that similarly to other tissues, neuronal cells develop insulin resistance and decrease glucose uptake—or in a more severe case almost completely switch to keton and fatty acid metabolism [129]. Amyloid-beta accumulation contributes to this metabolic switch by causing mitochondrial dysfunction and oxidative stress. Taken together, decreased O-GlcNAc modification on APP, intracellular hypoglycemia, and oxidative stress via continuous amyloid-beta deposition leads to neuronal degeneration [52, 130, 131].

### 4.5. Nuclear Transport

In eukaryotic cells, an important location of transport is at the double nuclear membrane. Bidirectional exchanges through this membrane are carried out by 125 × 10^6^ Da supramolecular complexes, called nuclear pore complexes (NPC) [132]. NPCs consist of various copies of ~30 different subunits called nucleoporins (Nups). Proteins below 40 kDa can pass through NPCs via passive diffusion, but protein transport above 40 kDa is an energy-dependent process. It requires the presence of nuclear localization signal (NLS) that is a sequence with high basic amino acid content exposed on the protein surface or a leucine-rich nuclear export signal (NES) [133]. O-GlcNAc has been suggested to interfere at two levels in the nuclear transport; it can modify proteins designated to nuclear translocation but may also alter the nucleoporins of nuclear pore complex [134].

First studies in 1989 assumed sugar residues can act as nuclear targeting signals [135]. Later, Duverger et al. performed experiments with fluorescein-coupled bovine serum albumin (BSA) in either electroporated or digitonin-permeabilized cells. According to their results, sugar-substituted BSA was able to enter the nucleus while control, unsubstituted albumin, stayed in the cytosol [136]. Subsequent studies also suggested the presence of an NLS-independent, sugar-mediated nuclear import of proteins [137, 138]. O-GlcNAc is likely to have remarkable influence on the nuclear transport and activity of beta-catenin. Besides participating in cell-cell adhesion, this protein also has a role in expression regulation as a transcriptional coactivator mediating *wnt* signaling. The latter function is involved in cell proliferation and invasion. O-GlcNAcylation of beta-catenin was demonstrated to have an inverse relationship with the protein's nuclear localization and transcriptional activity. Minimal O-GlcNAcylation of beta-catenin has been shown in tumor cells together with an elevated transcriptional state, while in normal cells, significantly, O-GlcNAcylated beta-catenin is associated with decreased transcriptional activity [31]. A central element in stress-related transcriptional regulation is NF*κ*B, which normally stays in the cytoplasm due to the inhibitory action of I*κ*B*α* which masks the NLS sequence of NF*κ*B [139]. Several studies showed that O-GlcNAc had a positive influence on NF*κ*B activation and nuclear translocation [48, 140, 141]. Multiple sites were found in the sequence of the p65 subunit to be directly O-GlcNAc modified, and data suggest that O-GlcNAc might disrupt/prevent the masking effect of I*κ*B*α* [48, 140]. Interestingly, Xing et al. found that O-GlcNAc modification has an opposite effect on NF*κ*B activation [33]. Other studies also elaborated on the inhibitory role of O-GlcNAc on NF*κ*B [72, 142]. This contradiction of results is difficult to resolve without more experimental data; however, the most plausible explanation would be that the interplay between several O-GlcNAc and phosphorylation sites on NF*κ*B has many variations. Depending on the cell type, the duration, type and severity of stress, and different posttranslational patterns may develop on NF*κ*B and produce different outcomes.

Nups were some of the first described OGT substrates, and they are among proteins with the highest O-GlcNAc density [143, 144]. As of now, 18 Nups have been identified as potential candidates for O-GlcNAc-modified proteins [145]. Despite emerging knowledge, the specific function of O-GlcNAc on Nups remains to be elucidated [26]. Recently, O-GlcNAc has been suggested to alter several structural and biophysical properties of NPCs and influence the interactions between soluble nuclear transport receptors (NTR) and Nups located at the central channel of NPCs, the so-called FG-Nups (phenylalanine-glycine-rich Nups) [146]. This way, O-GlcNAc may alter protein-protein interactions at the NPC thus modulating its permeability. Moreover, stability of FG Nups is also influenced by O-glycosylation via protection from ubiquitination and subsequent proteasomal degradation [145]. Thus, cross-talk between O-GlcNAcylation and ubiquitination also plays a role in stabilizing the NPC and maintaining the integrity of the selectivity filter.

### 4.6. Mitochondrial Transport

O-GlcNAcylation affects not only nucleocytoplasmic but also mitochondrial proteins as well by the help of mitochondrial (mOGT) and nucleocytoplasmic OGT (ncOGT) isoforms. The substrate for the modification, UDP-GlcNAc, is transferred into mitochondria via pyrimidine nucleotide carrier 1 (PNC1) [147, 148]. Only a few data is available regarding O-GlcNAc modification of specific mitochondrial carrier proteins. Mitochondrial permeability transition pore (mPTP) represents a nonspecific pore located in both the outer and inner mitochondrial membranes and allows molecules below 1.5 kDa to enter and exit the mitochondrial matrix. mPTP is activated by calcium overload and oxidative stress; its opening is a critical step in the initiation of apoptosis and cell death [63]. Reports demonstrated that elevated O-GlcNAc attenuates the mPTP opening [59, 149]. A central element of mPTP, voltage-dependent anion channel (VDAC), was revealed to be O-GlcNAc modified in cultured cardiac myocytes [59]. It was also demonstrated that cardiac mitochondria isolated from selective OGA-inhibited mice and OGT-overexpressing rat cardiomyocytes were resistant to the mPTP induction, while OGT inhibition increased sensitivity to Ca^2+^-induced mitochondrial swelling [59]. Hirose et al. also found supporting evidence for the protective effect of O-GlcNAc due to inhibition of mPTP opening; in their study, they used the anesthetic isoflurane for the preconditioning of cardiac myocytes and revealed that isoflurane increased O-GlcNAc modification of VDAC [150]. Another important player of mitochondrial permeabilization and apoptosis is Bcl-2, an antiapoptotic protein that inhibits mPTP opening possibly by direct interaction with VDAC. Bcl-2 is upregulated in association with hypoxic injury-induced cell death [151, 152]. Glucosamine treatment and OGT overexpression both significantly increased mitochondrial Bcl-2 levels under normoxic conditions and augmented the response to ischemia/reperfusion thus mediating the hyperglycemia-induced protective effect against hypoxic injury [64].

## 5. Conclusion and Perspectives

The number of proteins found to be O-GlcNAcylated is still rapidly increasing as of today. This is partly fueled by improvements in methodology, for example, by recent developments of mass spectrometry techniques (such as native mass spectrometry) or the availability of more potent and specific OGA and OGT inhibitors [153–155]. On the other hand, O-GlcNAc also received heightened interest in recent years; it is an excellent candidate for a direct signaling link between diverse cellular functions. A more practical reason of why O-GlcNAc deserves special attention is that it has a potential in human medicine. As mentioned above, O-GlcNAc plays a significant role in the development of AD and in diabetes, but disturbances of O-GlcNAc regulation are now considered in other syndromes such as malignant disorders or inflammatory diseases [33, 70, 156]. Recently, substantial research work has been dedicated to clarify its significance in hypoxia-induced or oxidative stress-related pathophysiological events [14, 63, 92]. Measurement of O-GlcNAc levels in human patients could report about important information. A few studies already attempted to use O-GlcNAc analysis to predict the extent of metabolic dysfunction and the complications of diabetes [157, 158]. On the other hand, intervening in O-GlcNAc regulation by specific OGT and OGA inhibitors could significantly improve the outcome of some diseases. For example, the protective effect of increased O-GlcNAc modification in ischemia/reperfusion experiments could be translated and utilized in human medicine in the future [79]. In malignant diseases, specific OGT inhibitors might support chemotherapy efforts by hindering insulin-independent glucose uptake or tilting the balance toward apoptosis in malignant cells [111].

What makes O-GlcNAc such a promising research subject—that is, its versatile nature—also makes it a difficult scientific endeavor. Since it influences so many different proteins and a wide variety of protein functions, general experimental approaches such as interfering with overall O-GlcNAc by altering the HBP metabolism or even by specific OGA/OGT inhibitors might lead to false results. The complexity that underlies this relatively simple mechanism is revealed by apparent paradoxes; for example, in acute stress situations, O-GlcNAc seems to be protective whereas chronic hyperglycemia-induced O-GlcNAc elevation clearly has a negative effect. In contrast, permanently decreased O-GlcNAc levels have been associated with AD, despite the fact that diabetic patients are more prone to the disease and that increased oxidative stress (which is supposed to elevate O-GlcNAc) is thought to contribute to the development of AD [159]. It seems to be that the analysis of both the spatial and temporal distribution of O-GlcNAc on individual proteins will be required for a complete understanding. Thus, studying O-GlcNAc on individual proteins is not only an option but it is also a necessity to locate specific regulatory events. In this review, our aim was to sum up the growing number of evidences supporting the idea that O-GlcNAc—directly or indirectly—influences membrane traffic elements. These data, taken together with the fact that O-GlcNAc is an important part of the cellular stress-adaptation mechanism, provides a firm basis for further studies to elucidate O-GlcNAc's role in the regulation of membrane transport under normal and pathological conditions as well.

## Figures and Tables

**Table 1:** O-GlcNAc-influenced membrane or membrane-associated transport proteins.

Protein	Function	Evidence for direct influence by O-GlcNAc	Evidence for indirect influence by O-GlcNAc	Reference
Potassium voltage-gated channel subfamily KQT member 3 (KCNQ3)	Voltage-dependent K^+^ channel	+	−	[97]
Potassium voltage-gated channel subfamily KQT member 2 and 5 (KCNQ2, KCNQ5)	Voltage-dependent K^+^ channel	MS hit	Form complex with KCNQ3	[93, 98]
Small conductance calcium-activated potassium channel protein 2 and 3 (KCNN2, KCNN3)	Voltage-independent calcium-activated K^+^ channel	MS hit	−	[93]
Calcium-activated potassium channel subunit alpha-1 (KCNMA1)	Calcium-activated K^+^ channel	MS hit	−	[93]
Potassium voltage-gated channel subfamily A member 4 (KCNA4)	Voltage-dependent K^+^ channel	MS hit	−	[93]
Voltage-gated sodium channels	Sodium transport	MS hit	Ankyrin G (link the cytoplasmic domains of integral proteins to cytoskeletal proteins)	[93, 100]
Sodium/potassium-transporting ATPase subunit alpha-2 and alpha-3	Plasma membrane sodium-potassium exchanger	MS hit	−	[93]
Stromal interaction molecule 1 (STIM1)	Ca^2+^ store depletion triggered Ca^2+^ influx in nonexcitable cells	+	−	[96]
Voltage-dependent calcium channels (CACNB3, CACNG3, CACNA1B, CACNA1A, CACNA1G)	Voltage-dependent Ca^2+^ channels in murine synapses	MS hit	−	[93]
Sarcoplasmic/endoplasmic reticulum calcium ATPase 2 (SERCA)	Endoplasmic Ca^2+^ reuptake. Playing a role in cardiac muscle contractility	+	Phospholamban (repressor of SERCA)	[94, 95]
Voltage-dependent anion-selective channel protein 1 and 3 (VDAC1, VDAC3)	Voltage-dependent anion channels in the outer mitochondrial and cell membrane	+	Bcl-2 (inhibits mPTP opening possibly by interaction with VDAC)	[59, 93, 151, 152]
Chloride channel-2 (ClC-2)	Cl^−^ channel	−	Sp1 (transcription factor)	[102]
Synapsin I	Regulation of synaptic vesicle release	+	−	[32, 114]
Alpha-synuclein	Regulation of neurotransmitter release	+	Identified O-GlcNAc sites influence its aggregation properties	[118]
Adaptor protein-1 (AP-1)	Endocytosis	−	Interaction with synergin gamma that has been identified as O-GlcNAc target	[37, 119, 120]
Piccolo (PCLO)	Cycling of synaptic vesicles	+	−	[116]
Amyloid-*β* precursor protein (APP)	Synapse formation	+	−	[22]
Solute carrier family 2, facilitated glucose transporter member 4 (GLUT4)	Glucose transporter	+	Munc18c (GLUT4 vesicle protein, influencing its translocation)	[109, 110]
Solute carrier family 2, facilitated glucose transporter member 1 (GLUT1)	Glucose transporter	−	HIF-1*α* (induces the transcription of GLUT1. O-GlcNAc affects the degradation of HIF-1*α*.)	[111]
Nucleoporins (Nups)	Nuclear transport	+	−	[26, 143–146]
Nup153	Nuclear transport for proteins and RNA	+	−	[160]
Importin *α*5	Nuclear transport receptor for proteins	−	Mediates the nuclear import of OGT	[37, 161]
Beta-catenin	Intracellular adhesion and transcriptional coactivator	+	−	[31]
Nuclear factor-*κ*B (NF*κ*B)	Transcriptional factor	+	−	[33, 48, 140, 141]

Specific proteins are selected and included in the table based on either direct, published evidence for O-GlcNAc modification, or indirect evidence (e.g., a molecular partner of the protein is known to be O-GlcNAcylated, or O-GlcNAc influences the expression of the protein). We considered finding O-GlcNAc protein only by mass spectrometry screening combined with various enrichment techniques (e.g., wheat germ agglutinin binding) as direct—albeit weak—evidence and indicated in the table as “MS hit.”
